# Systematic Analysis of miR-506-3p Target Genes Identified Key Mediators of Its Differentiation-Inducing Function

**DOI:** 10.3390/genes15101268

**Published:** 2024-09-27

**Authors:** Daniela F. Cardus, Mitchell T. Smith, Alexandra Vernaza, Jadyn L. Smith, Brynn Del Buono, Anupa Parajuli, Emma G. Lewis, Nakya Mesa-Diaz, Liqin Du

**Affiliations:** Department of Chemistry and Biochemistry, Texas State University, San Marcos, TX 78666, USAsmithm2152@gmail.com (M.T.S.); a_v651@txstate.edu (A.V.); xfq2@txstate.edu (J.L.S.); kqx5@txstate.edu (B.D.B.); cav193@txstate.edu (A.P.); egl44@txstate.edu (E.G.L.);

**Keywords:** miR-506-3p, neuroblastoma, miRNA targets, cell differentiation, neurite outgrowth

## Abstract

**Background/Objectives**: miR-506-3p has been demonstrated to be a strong inducer of neuroblastoma cell differentiation, highlighting the potential of applying miR-506-3p mimics to neuroblastoma differentiation therapy. However, the target genes of miR-506-3p that mediate its differentiation-inducing function have not been fully defined. This study aims to comprehensively investigate the targetome of miR-506-3p regarding its role in regulating neuroblastoma cell differentiation. **Methods**: We combined gene expression profiling and functional high-content screening (HCS) to identify miR-506-3p target genes that have differentiation-modulating functions. For evaluating the potential clinical relevance of the identified genes, we analyzed the correlations of gene expressions with neuroblastoma patient survival. **Results**: We identified a group of 19 target genes with their knockdown significantly inducing cell differentiation, suggesting that these genes play a key role in mediating the differentiation-inducing activity of miR-506-3p. We observed significant correlations of higher mRNA levels with lower patient survival with 13 of the 19 genes, suggesting that overexpression of these 13 genes plays important roles in promoting neuroblastoma development by disrupting the cell differentiation pathways. **Conclusions**: Through this study, we identified novel target genes of miR-506-3p that function as strong modulators of neuroblastoma cell differentiation. Our findings represent a significant advancement in understanding the mechanisms by which miR-506-3p induces neuroblastoma cell differentiation. Future investigations of the identified 13 genes are needed to fully define their functions and mechanisms in controlling neuroblastoma cell differentiation, the understanding of which may reveal additional targets for developing novel differentiation therapeutic agents.

## 1. Introduction

Neuroblastoma is one of the most prominent extracranial solid tumors that arises from neural crest precursor cells that fail to complete the differentiation process [1,2]. Under pathological developmental conditions, incomplete differentiation of neural crest precursor cells form neuroblastomas within the sympathetic nervous system [1,2]. Neuroblastomas mainly occur in children under 15 years old and account for 15% of pediatric cancer-related deaths [3,4,5]. Because tumorigenesis of neuroblastomas occurs due to a deficiency of cell differentiation, differentiation-inducing agents have been applied to neuroblastoma treatment. Indeed, treatment of high-risk neuroblastomas with differentiation agents such as retinoic acids have been proven to be beneficial to a subset of high-risk neuroblastoma patients [6,7]. However, the response rate of neuroblastoma patients to retinoic acids is not ideal; more than 50% of the high-risk neuroblastomas treated with retinoic acids still develop recurrence, indicating that at least a subset of the neuroblastoma cell populations in these neuroblastomas are resistant to retinoic acid treatment. Lack of understanding of the differentiation mechanisms in neuroblastoma cells has been a major obstacle to developing alternative differentiation agents for patients that are not responsive to retinoic acids.

microRNAs (miRNAs) are a class of endogenously expressed small RNAs that regulate expression of target genes by complementary binding to the 3′ untranslated regions (3′UTR) of mRNAs [8]. The critical roles of miRNAs in tumorigenesis have been well established [9]. In addition, synthetic miRNA mimics that simulate the functions of tumor-suppressive miRNAs have shown potential to treat cancers [10,11]. Recently, a group of miRNAs that have strong functions in inducing neuroblastoma cell differentiation has been identified [12]. This indicates that miRNAs represent a key molecular mechanism that controls the cell differentiation process in neuroblastomas and suggests the promise of applying miRNA mimics to differentiation therapy. Among the differentiation-inducing miRNAs identified in neuroblastoma cells, miR-506-3p has been shown to have the highest differentiation-inducing activity in multiple neuroblastoma cells with distinct genetic backgrounds [12], suggesting that developing miR-506-3p-based differentiation therapy would potentially benefit a large number of neuroblastoma cases. However, the mechanisms of action of miR-506-3p in inducing neuroblastoma cell differentiation have not been fully characterized. Full characterization of its mechanisms of action would help to comprehensively evaluate the potential therapeutic benefits and side effects of miR-506-3p for treating neuroblastoma patients. Towards this end, the first step is to identify the direct target genes of miR-506-3p that have a role in regulating neuroblastoma cell differentiation. Several direct target genes of miR-506-3p have been reported to play a role in mediating the differentiation-inducing function of miR-506-3p [12,13]. However, results from these studies showed that these recognized targets are only responsible for a portion of the differentiation-inducing activity of miR-506-3p, suggesting that there are unknown targets of miR-506-3p that play important roles in its differentiation-inducing mechanism.

In this study, we intend to systematically investigate the targetome of miR-506-3p regarding its role in regulating neuroblastoma cell differentiation. We combined informatics, gene expression array, and HCS analyses to investigate the role of miR-506-3p target genes in modulating neuroblastoma cell differentiation. For the identified targets, we further investigated their potential clinical relevance by analyzing the correlation of gene expression with neuroblastoma patient survival using public neuroblastoma patient datasets.

## 2. Materials and Methods

**Cell lines and materials.** The human neuroblastoma cell line BE(2)-C was obtained from American Type Culture Collection (Manassas, VA, USA. Cat#, CRL-2268). We chose BE(2)-C for this study because this cell line is highly suitable for high-throughput quantification of neurite outgrowth [12,14], which is needed for the HCS of the miR-506-3p target genes. Cells were cultured in Dulbecco’s Modified Eagle Medium (DMEM)/F12 (Corning Inc., Corning, NY, USA. Cat#, MT10090CV) with 10% FETALGRO bovine growth serum (FBS) (Fisher Scientific, Waltham, MA, USA. Cat#, NC1928574) and 1% penicillin–streptomycin (P/S) (Fisher Scientific. Cat#, MT30001CI). miR-506-3p mimic (leading sequence, UAAGGCACCCUUCUGAGUAGA; carrier sequence, UACUCAGAAGGGUGCCUUAUU) and negative control oligonucleotides (oligos) (leading sequence, AUUCCGUGCCUUCUGAGUAGA; carrier sequence, UACUCAGAAGGCACGGAAUUU) were purchased from Sigma Aldrich (St Louis, MO, USA). siRNAs were purchased from Horizon Discovery (Cambridge, United Kingdom). Invitrogen™ Lipofectamine™ RNAiMAX transfection reagent was purchased from Fisher Scientific (Cat#, 13-778-150). MTT (2-(4,5—Dimethylthiazol-2-yl)-2,5-Diphenyltetrazolium Bromide) reagent was purchased from Fisher Scientific (Cat#, AC15899).

**Quantitative RT-PCR (qRT-PCR).** Total RNA was isolated from neuroblastoma cells using the Invitrogen™ miRVana miRNA isolation kit (Fisher Scientific. Cat#, A1560) as previously reported [15]. The kit can be used for either total RNA isolation or miRNA-enriched small-RNA isolation. We used it to isolate total RNAs. Briefly, the collected cell pellet was first lysed using the lysis buffer (provided by the kit); then, Acid-Phenol:Chloroform (provided by the kit) was used to separate total RNA into the aqueous phase. Finally, ethanol was used to precipitate total RNA from the aqueous solution using a column-based precipitation approach (the column was provided by the kit). A sample of 2 µg RNA was reverse transcribed into cDNA using the Applied Biosystems^TM^ High-Capacity cDNA Reverse Transcription Kit (Fisher Scientific. Cat#, 43-688-14). miR-506-3p expression was measured by qPCR using the Applied Biosystems^TM^ TaqMan microRNA Assays (Fisher Scientific. Cat#, 10524815) with expression of RNU44 RNA used as a loading control. Threshold cycle times (C_t_) were obtained, and relative gene expression was calculated using the comparative cycle time method. Briefly, for the comparative cycle time method, normalization of each treatment (C_t_x) to the loading control (C_t_l) was calculated by subtraction (ΔC_t_ = C_t_x − C_t_l). The second subtraction was calculated to obtain the ΔΔCt for each experimental treatment (e.g., miR-506-3p mimic treatment) by subtracting the ΔCt of the negative control oligo-treated cells (ΔC_t_n) from the ΔCt of each experimental treatment (ΔΔC_t_ = ΔC_t_ − ΔC_t_n). Since each cycle of the PCR reaction equaled a two-fold difference in copy number of the amplified cDNA, the fold change of expression associated with each experimental treatment was calculated by 2^−ΔΔC^_t_.

**mRNA expression array**. BE(2)-C cells were transfected with 20 nM of miR-506-3p mimic or negative control oligos in 100 mm cell culture dishes. After 24 h, total RNA was isolated as above. mRNA expression profiling was performed using the Illumina mRNA WG-6 v4 microarray platform, and data were analyzed using the approach as previously reported [12].

**Transfection with siRNAs, miR-506-3p, and negative control oligos.** To enable the reverse transfection process, 5 uL of the siRNAs or controls were added to 96-well plates, and then 15 uL of diluted Lipofectamine RNAiMAX was added, mixed with the oligos, and incubated for a duration of 5 min at room temperature. Throughout this time, RNAiMAX-oligo micelles complexes were formed. Specified amounts of BE(2)-C cells (2000 cells per well in 100 µL serum-free, antibiotics-free culture media) were seeded into 96-well plates. The cells were then cultured at 37 °C overnight to allow for the transfection of the oligos into the cells. On the second day, 80 µL of DMEM/F-12 Cell Culture Media supplemented with 10% FBS and 1% P/S was added to each well. The cells were then cultured at 37 °C for 3 additional days before neurite length and cell viability assays were performed. In total, the cells were cultured for 4 days after transfection. We chose 4 days of transfection treatment because our previous publication indicated that 4 days are sufficient to reach the maximum neurite outgrowth and cell growth arrest induced by differentiation-inducing treatments [12].

**siRNA-based HCS of neurite outgrowth.** BE(2)-C cells were transfected with 20 nM of the 283 siRNA library in four replicates in 96-well plates as above. We chose to treat cells with siRNAs at 20 nM because our published results showed that 20 nM is close to the minimum concentration for differentiation-inducing siRNAs to reach their maximum effect in inducing neurite outgrowth and cell growth arrest [16]. For detecting neurite outgrowth after four days of transfection, plates were placed into the ZOOM IncuCyte Imaging System (Essen BioScience, Ann Arbor, MI, USA) and cell images were taken under 20X microscopic magnification. The neurite lengths associated with each treatment were measured using the neurite definition that was pre-defined with a set of images of differentiated BE(2)-C cells using the neurite definition function of the NeuroTrack system (Essen BioScience). Because the undifferentiated BE(2)-C has a basal neurite length value and this basal neurite length is very sensitive to slight cell confluence variation between plates, internal normalization within each plate is needed to allow for plate-to-plate comparison. Therefore, neurite length associated with each well in each plate was first internally normalized to the mean of the corresponding plate, and multiple screen plates were then aggregated together to allow for comparison of each siRNA to the library mean to calculate the fold change of neurite outgrowth associated with each siRNA. The scatter plot of neurite outgrowth from the HCS was generated using the Prism GraphPad software (version 7.05).

**MTT assay to measure cell survival and proliferation.** The cell survival and proliferation after different treatments were evaluated by measuring and comparing the amounts of viable remaining cells in each well at the end of the treatment course using the MTT assay as before [17]. Cells were plated in 96-well plates and treated as described above. For the MTT assay, 100 µL of the MTT solution (0.25 mg/mL in cell culture medium) was added to each well and incubated for 2 h at 37 °C. The formazan crystals, products of the MTT reaction, were then centrifuged down at 1400 rpm for 5 min. After centrifugation, the supernatant was removed, and the precipitates were dissolved in 100 µL of dimethyl sulfoxide (DMSO) by incubating at 37 °C for 5 min. The absorbances at 630 nm (A_630_) and 570 nm (A_570_) were measured, and the difference in the two absorbance values (ΔA = A_630_ − A_570_) represents the specific absorbance of the formazan product and, therefore, reflected the number of viable cells in each well. The relative cell viability was determined by normalizing the absorbance value associated with each treatment (ΔA_T_) to the absorbance value associated with the negative control treatment (ΔA_C_).

**Neuroblastoma patient survival analysis**. The analyses were performed to examine the clinical relevance of miR-506-3p target gene expression in neuroblastoma tumors based on two published neuroblastoma patient datasets in the R2 Genomics (R2) platform (http://r2.amc.nl, accessed on 1 June 2022) and the Kocak and SEQC (the seqcnb1 platform) datasets [18]. The access to R2 datasets is free, and patient survival analysis can be conducted directly on the website using the Kaplan–Meier method. Specifically, the patients in each dataset were separated into two groups based on the mRNA levels of a specific gene in tumor specimens, the low- and high-expression groups, by running the R2 Scan mode based on the overall survival (OS) data. The Scan-mode analysis sorts the tumor mRNA levels of the gene from low to high, and then runs every possible low- and high-group classification (i.e., for a dataset that has 500 patients, the first possible two-group classification is that the low-expression group contains 1 patient, and the high-expression group contains 499 patients, and so on) and finds the grouping that gave the maximum separation (i.e., lowest *p* value) of the OS curves between the low- and high-expression groups. The statistical significance of OS between the two groups was determined by 2-tailed log-rank tests, with raw *p* < 0.01 considered as statistically significant. The grouping was then implemented into the event-free survival (EFS) analysis curves for consistency.

**miR-506-3p target identification**. The targets of miR-506-3p were identified using the microRNA Target Filter function of the Ingenuity Pathway Analysis (IPA) platform. The microRNA Target Filter function of IPA identifies all experimentally validated and predicted targets for a specific miRNA by accessing several high-impact miRNA databases, including TargetScan, TarBase, and miRecords.

**Other statistical analysis**. Statistical analysis of differences in gene expression levels between patient groups was performed using the two-tailed Student’s T-test as previously reported [19,20], with *p* < 0.05 considered as statistically significant. For the neurite outgrowth and cell viability data from the HCS, the normality of the data distribution was analyzed using the Shapiro–Wilk test, with *p* < 0.05 considered as not passing the normality test. Since both the neurite outgrowth and cell viability data distributions failed the normality tests, the statistical significance of the effect of the siRNAs on neurite outgrowth and cell viability was examined by comparing to the corresponding mean values of the whole siRNA library using nonparametric Mann–Whitney U tests, with *p* < 0.05 considered statistically significant. For all other experiments, the statistical significance of the comparison between each treatment and the control group was determined by two-tailed Student’s *t*-test as previously reported [12,19,20], with *p* < 0.05 considered statistically significant.

## 3. Results

### 3.1. Expression Array Analysis Identified miR-506-3p Target Genes with Their mRNA Levels Down-Regulated by miR-506-3p Mimic

**Figure 1:**
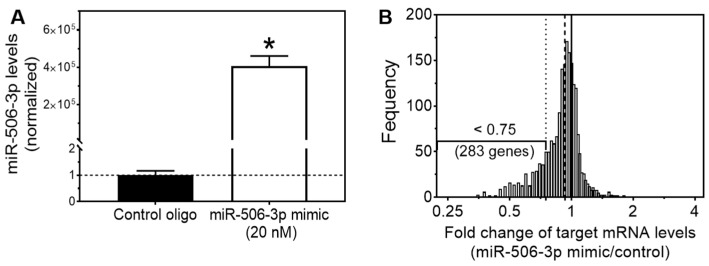
Effect of miR-506-3p overexpression on mRNA levels of its predicted targets. BE(2)-C cells were transfected with 25 nM miR-506-3p mimic or control for 24 h. mRNA expressions were measured by Illumina v4 array. (**A**) miR-506-3p levels in BE(2)-C cells transfected with miR-506-3p mimic and negative control oligo as measured by qRT-PCR. *. *p* < 0.05 compared to the control group. (**B**) Histogram of changes in mRNA levels of the 1942 predicted targets induced by miR-506-3p mimic, expressed as the ratio of miR-506 mimic treatment vs. control oligo (miR-506 mimic/control). The solid vertical line marks the ratio of 1.00, which is the mean of the normalized signals of all mRNAs measured in the Illumina array platform. The dashed vertical line represents the median ratio of the 1942 targets of miR-506-3p, which equals 0.93. The dotted vertical line marks the 0.75 (i.e., mRNA level was downregulated by 0.25) cut-off that we used in this study.

In order to identify the predicted direct target genes of miR-506-3p, we applied IPA to identify genes that contain the predicted miR-506-3p binding sites in the 3′UTR of mRNAs by analyzing all known human protein-coding genes. The prediction was based on base pairing of the miR-506-3p seed sequence region with the 3′UTR region of a given mRNA [8,21]. The analysis yielded 1942 genes with their mRNA products containing one or more miR-506-3p binding sites in their 3′UTRs. In order to determine whether these predicted targets were truly regulated by miR-506-3p, we exploited gene expression analysis to systematically investigate whether miR-506-3p overexpression would down-regulate mRNA levels of the 1942 target genes in a neuroblastoma cell line BE(2)-C. Figure 1A shows that cellular miR-506-3p level was significantly higher in cells transfected with miR-506-3p mimic compared to cells transfected with negative control oligo, confirming successful overexpression of miR-506-3p in cells by miR-506-3p mimic. The results of the expression array analysis (Figure 1B) showed that more of the 1942 predicted mRNA targets were dramatically downregulated in expression than upregulated in expression by miR-506-3p mimic, as indicated by the left-shift of the median value (dashed line, 0.93) from the mean (solid line, 1.00), which demonstrates that the majority of the predicted miR-506-3p targets were its true biological targets and were effectively downregulated by miR-506-3p mimic at mRNA levels. Further analysis identified that 283 of the 1942 target genes were down-regulated by >25% by the miR-506-3p mimic. We decided to focus on investigating target genes that were down-regulated by >25%, because previous findings supported that down-regulation of miRNA targets at mRNA levels by 25% would be sufficient to lead to a biologically meaningful phenotype [15].

### 3.2. HCS Identified Target Genes of miR-506-3p with Their Knockdown by siRNAs Significantly Inducing Neurite Outgrowth in Neuroblastoma Cells

**Figure 2:**
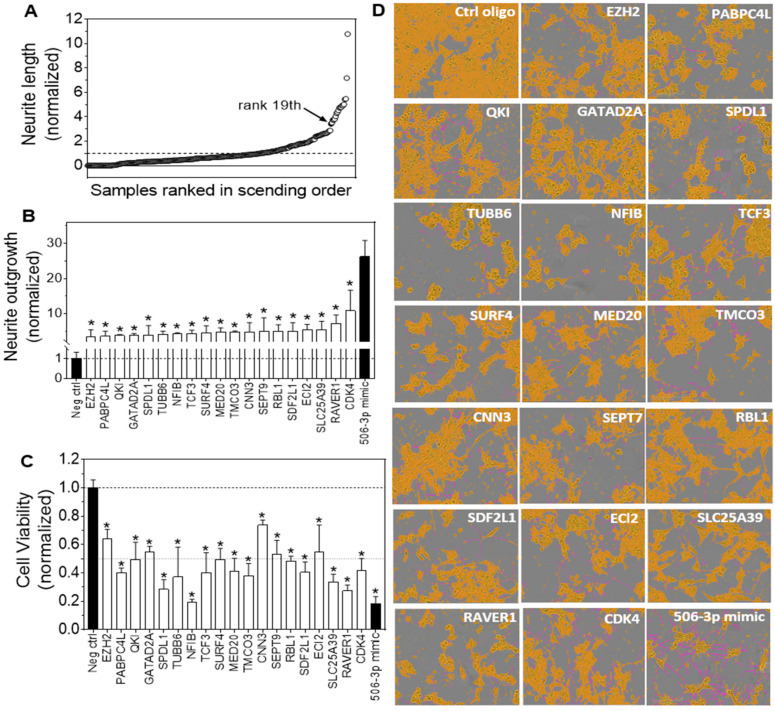
Effect of knocking down miR-506-3p target genes on neurite outgrowth and cell viability in BE(2)-C cells. Cells were transfected with 20 nM of siRNAs, miR-506-3p mimic, or negative control oligos. After 4 days, cell images were taken, and neurite lengths were quantified using IncuCyte Zoom. After the cell images were taken, cell viability was determined by MTT as described in Materials and Methods. (**A**) Scatter plot of normalized neurite lengths, sorted in ascending order, associated with individual siRNAs. (**B**) Effect of siRNAs against the indicated genes on neurite outgrowth. *. *p* < 0.05, significantly lower compared to miR-506-3p mimic. (**C**) Effect of siRNAs against the indicated treatment on cell viability. *. *p* < 0.05, significantly decreased compared to the negative control oligo (Neg ctrl). (**D**) Cell images showing the effect of miR-506-3p mimic and siRNAs against individual genes on neurite outgrowth. Shown are the representative phase-contrast images defined for cell body areas (yellow) and neurites (pink).

In order to determine whether the 283 targets identified above play a role in mediating the differentiation-inducing activity of miR-506-3p, we exploited an HCS approach to investigate whether knocking down mRNA expression using siRNA of each gene would induce neurite outgrowth, the morphological differentiation marker of neuroblastoma cells, in BE(2)-C cells. The scatter plot shown in Figure 2A indicates that the effects of knocking down the expression of the 283 genes on neurite outgrowth vary greatly; the results show that a group of 19 genes were clustered together, with the neurite outgrowth associated with siRNAs against these 19 genes clearly separated from and higher than the rest of the 283 genes. The clear separation of the 19 genes indicates that knocking down each of these 19 genes had much more potent differentiation-inducing effects than the rest of the 283 genes, which therefore supports that they play a more important role in modulating cell differentiation and in mediating the differentiation-inducing activity of miR-506-3p than the rest of the target genes. Figure 2B compares the neurite outgrowth induced by miR-506-3p mimic and by individual knockdown of the 19 genes. The results indicate that knocking down each of the 19 genes, including enhancer of zeste 2 polycomb repressive complex 2 subunit (*EZH2*), poly(A) binding protein cytoplasmic 4 like (*PABPC4L*), KH domain-containing RNA binding (*QKI*), GATA zinc finger domain-containing 2A (*GATAD2A*), spindle apparatus coiled-coil protein 1 (*SPDL1*), tubulin β 6 (*TUBB6*), nuclear factor I B (*NFIB*), transcription factor 3 (*TCF3*), surfeit 4 (*SURF4*), mediator complex subunit 20 (*MED20*), transmembrane and coiled-coil domains 3 (*TMCO3*), calponin 3 (*CNN3*), Septin 9 (*SEPT9*), retinoblastoma-like protein 1 (*RBL1*), stromal cell-derived factor 2 like 1 (*SDF2L1*), Enoyl-CoA Delta Isomerase 2 (*ECI2*), solute carrier family 25 member 39 (*SLC25A39*), Ribonucleoprotein PTB-binding 1 (*RAVER1*) and cyclin-dependent kinase 4 (*CDK4*), dramatically induced neurite outgrowth compared to the negative control oligo. However, the neurite-inducing effect of knocking down each individual gene was significantly lower than the effect of miR-506-3p mimic, indicating that the differentiation-inducing activity of miR-506-3p is a combined action of a cohort of its target genes. Correspondingly, Figure 2C shows that knocking down each of the 19 genes significantly reduced the number of viable cells compared to the control as measured by MTT assay, which demonstrated that the neurite outgrowth induced by gene knockdown was accompanied by cell growth arrest. These results further support that cell differentiation is truly induced by knocking down expression of these genes. Figure 2D shows the representative cell images associated with the 19 siRNAs and miR-506-3p mimic, which further demonstrate that the neurite outgrowth induced by gene knockdown is accompanied by reduced cell proliferation.

Table 1 shows the miR-506-3p target sites and sequences in the 3′UTRs of the 19 mRNAs. Our experimental results presented above altogether strongly support that these 19 genes are true target genes of miR-506-3p and that, together, they play a key role in mediating the differentiation-inducing function of miR-506-3p in neuroblastoma cells.

### 3.3. Neuroblastoma Patient Survival Analysis Shows That 13 of the 19 Target Genes Exhibit Oncogenic Potential

**Figure 3:**
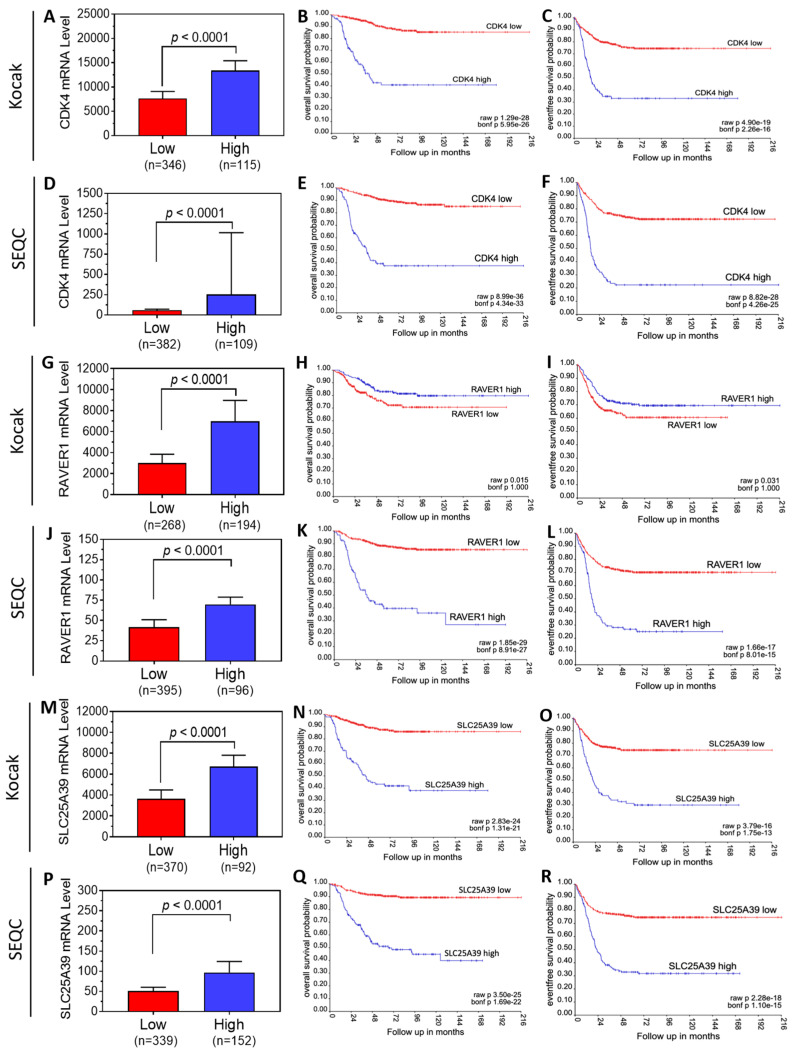
**Correlation of tumor mRNA levels with neuroblastoma patient survival for *CDK4*, *RAVER1*, and *SLC25A39*.** (**A**–**F**), Correlation of tumor *CDK4* mRNA levels with patient survival in the Kocak (**A**–**C**) and SEQC (**D**–**F**) datasets. Patients in the two datasets were grouped into low and high groups based on the tumor *CDK4* mRNA levels (**A**,**D**) and comparison of OS (**B**,**E**) and EFS (**C**,**F**) curves between the low and high expression groups in each dataset using Kaplan–Meier survival analysis. (**G**–**L**), Correlation of tumor *RAVER1* mRNA levels with patient survival in the two datasets. (**M**–**R**), Correlation of tumor *SLC25A39* mRNA levels with patient survival in the two datasets.

Focusing on the top 19 target genes identified above, we investigated their oncogenic potential by analyzing the correlations of their mRNA levels with neuroblastoma patient survival in two public patient datasets from the R2 Genomics platform, the Kocak and the SEQC datasets. Figure 3 shows the Kaplan–Meier OS and EFS curves in both datasets for the top 3 genes. Patients in each dataset were grouped into low- and high-expression groups based on tumor mRNA levels of each gene, as described in the Materials and Methods section. The oncogenic potential of a specific gene was identified based on a statistically significant (i.e., raw *p* < 0.01) separation of survival curves, with patients in the high expression group exhibiting lower patient survivability compared to patients in the low expression group.

Figure 3A–F show the correlation of *CDK4* mRNA expression with patient survival in both datasets. As shown in Figure 3A,D, *CDK4* mRNA levels between the high- and low-expression groups were significantly different in each dataset. Kaplan–Meier survival analyses showed that patients in the high *CDK4* expression group exhibited both significantly lower OS (Figure 3B,E) and lower EFS (Figure 3C,F) compared to patients in the low group in both datasets, supporting the potential oncogenic function of CDK4 to promote tumor progression and to drive poorer prognosis in neuroblastoma patients. Conversely, *RAVER1*, the second gene that was identified from HCS, demonstrated slightly, but not significantly, higher OS and EFS in the high-*RAVER1*-expression group compared to the low-expression group in the Kocak dataset (Figure 3G–I). In the SEQC dataset, however, the high-*RAVER1*-expression group exhibited significantly lower OS (Figure 3K) and lower EFS (Figure 3L), supporting its oncogenic potential. Due to the inconsistency of the results from the two datasets, a conclusion regarding the clinical relevance of this gene cannot be drawn.

The third gene, *PABPC4L*, displayed dramatically and significantly lower OS and EFS in patients with high *PABPC4L* expression levels compared to patients with low expressions in both datasets (Figure 3M–R), strongly supporting its oncogenic potential. To allow for an overall comparison of all 19 genes identified from HCS, a summary of the survival analysis from the Kocak and SEQC datasets is provided in Table 2. The oncogenic potential of a specific gene was determined based on the following criteria: (1) the gene exhibits lower OS and EFS in the high-mRNA-expression group compared to the low-expression group in all the available datasets; and (2) a majority of the raw *p* values (e.g., 3 of the 4 raw *p* values), if not all, for the gene reach *p* < 0.01. Based on these criteria, 13 of the 19 genes were identified as having oncogenic potential, as shown in Table 2. Together with our experimental findings, our findings strongly suggest that the differentiation-inducing targetome of miR-506-3p is highly enriched with oncogenic genes that play an important role in determining neuroblastoma progression and patient prognosis.

## 4. Discussion

Through investigating the direct targetome of miR-506-3p, we identified 19 genes with their knockdown significantly and dramatically inducing neuroblastoma cell differentiation. The results suggest that, altogether, they play a key role in mediating the differentiation-inducing function of miR-506-3p. More importantly, by analyzing the correlations of the tumor mRNA levels of the 19 genes with neuroblastoma patient survival, we identified that 13 genes exhibit oncogenic potential, with their high mRNA levels significantly correlated with poorer patient survival probability. These findings strongly support the clinical relevance of the miR-506-3p and its targeted genes in neuroblastoma tumorigenesis—down-regulation of miR-506-3p expression or up-regulation of expression of the 13 oncogenic targets may be key factors that drive tumor progression and lead to poorer patient prognosis. Our findings altogether provide a strong rationale for miR-506-3p mimic-based therapy—miR-506-3p mimic would downregulate expression of its oncogenic targetome, which would further block tumor progression and improve patient prognosis.

Among the 13 oncogenic target genes that we identified, only 2 genes, *CDK4* and *EZH2*, have been clearly demonstrated to have a strong function in modulating neuroblastoma cell differentiation, with knocking down expression of these 2 genes inducing neuroblastoma cell differentiation [12,13], which is consistent with the previous findings. However, the molecular mechanisms by which these two genes regulate neuroblastoma cell differentiation have not been fully elucidated. The remaining 11 genes have not been found to regulate neuroblastoma cell differentiation previously. However, five of the 11 genes, including calponin (*CNN3*), *GATAD2A*, *NFIB*, *TCF3*, and *RBL1*, have been suggested to play a role in modulating cell differentiation in other cell types. *CNN3* plays a role in cytoskeletal reorganization and has been reported to function in cell differentiation, proliferation, and migration [22]. *GATAD2A* functions as a component of the Nucleosome Remodeling and Deacetylase (*NuRD*) complex, which has been shown to play a role in the cell differentiation processes [23]. *NFIB* is a transcription factor that has been shown to have multiple biological functions, including modulating cell differentiation and maturation during embryonic development [24,25]. *RBL1* has been shown to play a role in modulating endoderm differentiation of human embryonic stem cells [26]. *TCF3* is an important transcription factor for mediating cell proliferation and activation in the Wnt signaling pathway [27,28], a pathway that has been known to regulate cell self-renewal and differentiation [29]. Our work identified the roles of these five genes in controlling neuroblastoma cell differentiation for the first time, extending the differentiation-regulating functions of these genes to neuroblastoma. The remaining six genes, *MED20*, *PABPC4L*, *SDF2L1*, *SLC25A39*, *SPDL1*, and *SURF4*, have not been linked to cell differentiation. Overall, 11 of the 13 differentiation-regulating target genes of miR-506-3p that we identified represent our novel findings. Further investigations into these genes are certainly warranted in the future. Investigating the identified 13 differentiation-modulating genes as a group by combining in silico and in vitro approaches would be an efficient way to elucidate their interactive network and their mechanisms of action in regulating neuroblastoma cell differentiation, which may reveal addition targetable genes and pathways for developing novel approaches to differentiation therapy.

It is worth noting that 2 of 19 genes, *ECI2* and *TMCO3*, clearly exhibited tumor-suppressive potential in neuroblastoma patients, with their low expressions correlated with poorer patient survival in both datasets. This indicates the complexity of the cellular regulatory network mediated by miR-506-3p in neuroblastoma cells. This is not surprising given the large number of targets genes of miR-506-3p (total of 1942 predicted target genes). However, this does not diminish the potential value of miR-506-3p mimic-based therapy because: (1) a majority of the 19 genes are oncogenic, which supports that the overall impact of its targetome downregulation induced by miR-506-3p mimic would be tumor-suppressive; (2) we have experimentally observed the tumor-suppressive effect of miR-506-3p mimic in neuroblastoma cells, as demonstrated by the reduced cell proliferation and induced cell differentiation following miR-506-3p treatment. In addition, this anti-suppressive impact exerted by the downregulation of the two tumor-suppressive targets might also be beneficial. It might be an important mechanism to prevent the over-anti-proliferative impact on normal cells when miR-506-3p mimic is used for anti-cancer therapy. The non-cancer cells in normal tissues might use this mechanism to fight against the anti-proliferative function of miR-506-3p. The potential therapeutic benefits and potential side effects of miR-506-3p mimic-based therapy certainly need to be fully evaluated in in vivo investigations in the future. For the remaining 4 of the 19 genes, *QKI*, *RAVER1*, *SEPT9*, and *TUBB6*, their correlations with patient survival were not consistent between the two datasets, with the results from one dataset suggesting their tumor-suppressive potential and the other dataset supporting oncogenic potential, although several correlations did not reach statistical significance. Further studies of these genes in larger neuroblastoma patient populations are needed to clearly define their functions in neuroblastoma tumorigenesis.

In this study, we only investigated the 283 target genes of miR-506-3p that are downregulated by >25% by miR-506-3p mimic due to the limited scope of this study. The >25% is an arbitrary cutoff that we established based on the literature [15], and we assumed that target genes that were downregulated more dramatically by miR-506-3p mimic would play more important roles in mediating its differentiation-inducing function. It is plausible to speculate that certain target genes that are downregulated by ≤25% by miR-506-3p mimic play non-neglectable roles in mediating its differentiation-inducing function, and we will further investigate these targets in the future.

## 5. Conclusions

In conclusion, our study identified a group of novel differentiation-regulating target genes of miR-506-3p that exhibit potential clinical significance in determining neuroblastoma progression and patient survival, which represents a significant advancement in understanding the differentiation-inducing and tumor-suppressive mechanisms of miR-506-3p. These findings strongly support the potential therapeutic benefit of miR-506-3p mimic.

## Figures and Tables

**Table 1:** The predicted miR-506-3p targets sites in the 3′UTRs for the 19 target genes.

(1) Rank	(2) Name	(3) Target Site:miRNA Sequence	(4) Seed Position
1	CDK4 3′ UTR	5′ AGAGAUUACUUUGC**UGCCUUA** 3′	355–362
	hsa-miR-506-3p	3′ AGAUGAGUCUUCCC**ACGGAAU** 5′	
2	RAVER1 3′ UTR	5′ CCCUCCCCUUCUGA**UGCCUUA** 3′	648–655
		5′ CGCUGAAACCCUGCA**GCCUUA** 3′	226–232
	hsa-miR-506-3p	3′ AGAUGAGUCUUCCC**ACGGAAU** 5′	
3	SLC25A39 3′ UTR	5′ GAGACCCAGCCAAG**UGCCUU**U 3′	69–75
	hsa-miR-506-3p	3′ AGAUGAGUCUUCCC**ACGGAAU** 5′	
4	ECI2 3′ UTR	5′ AAUAAGCUUCAUUG**UGCCUU**U 3′	87–93
	hsa-miR-506-3p	3′ AGAUGAGUCUUCCC**ACGGAAU** 5′	
5	SDF2L1 3′ UTR	5′ UAGGGGUCCUCAAG**UGCCUU**U 3′	92–98
	hsa-miR-506-3p	3′ AGAUGAGUCUUCCC**ACGGAAU** 5′	
6	RBL1 3′ UTR	5′ AGGAAUAUUUUAAG**UGCCUU**U 3′	210–216
		5′ CUCACCCCUUCUCG**UGCCUU**U 3′	838–844
	hsa-miR-506-3p	3′ AGAUGAGUCUUCCC**ACGGAAU** 5′	
7	SEPT9 3′ UTR	5′ CCUGGAGCAGAAAG**UGCCUU**U 3′	626–632
	hsa-miR-506-3p	3′ AGAUGAGUCUUCCC**ACGGAAU** 5′	
8	CNN3 3′ UTR	5′ CUUUUAAGAAAAAU**UGCCUUA** 3′	127–133
	hsa-miR-506-3p	3′ AGAUGAGUCUUCCC**ACGGAAU** 5′	
9	TMCO3 3′ UTR	5′ UGUGGUGCCUGGAU**GUGCCU**U 3′	707–713
	hsa-miR-506-3p	3′ AGAUGAGUCUUCCC**ACGGAAU** 5′	
10	MED20 3′ UTR	5′ GCUGUUUUACUCCG**UGCCUUA** 3′	213–220
	hsa-miR-506-3p	3′ AGAUGAGUCUUCCC**ACGGAAU** 5′	
11	SURF4 3′ UTR	5′ UUUACAAUUUGUGA**UGCCUUA** 3′	1344–1350
		5′ AAGUUUUCUAACAC**UGCCUUA** 3′	1391–1397
	hsa-miR-506-3p	3′ AGAUGAGUCUUCCC**ACGGAAU** 5′	
12	TCF3 3′ UTR	5′ AGAGAAGAAAAAAA**UGCCUUA** 3′	394–401
	hsa-miR-506-3p	3′ AGAUGAGUCUUCCC**ACGGAAU** 5′	
13	NFIB 3′ UTR	5′ ACUGACUUUCUAGA**UGCCUUA** 3′	312–319
	hsa-miR-506-3p	3′ AGAUGAGUCUUCCC**ACGGAAU** 5′	
14	TUBB6 3′ UTR	5′ UUCUUGAACCCUGG**UGCCU**GU 3′	61–65
	hsa-miR-506-3p	3′ AGAUGAGUCUUCCC**ACGGAAU** 5′	
15	SPDL1 3′ UTR	5′ CUGGCAUUUUCAUG**UGCCUU**U3′	586–592
	hsa-miR-506-3p	3′ AGAUGAGUCUUCCC**ACGGAAU** 5′	
16	GATAD2A 3′ UTR	5′ GCAAAAGUGUGAGA**UGCCUUA** 3′	2316–2323
	hsa-miR-506-3p	3′ AGAUGAGUCUUCCC**ACGGAAU** 5′	
17	QKI 3′ UTR	5′ UAAAGAAAAGAAAG**UGCCUUA** 3′	8119–8125
		5′ UUGUAGUUUUAAAA**UGCCUUA** 3′	6081–6087
		5′ AUUCACAUCUCCUC**UGCCUUA** 3′	5685–5691
		5′ UUUUAAAACUACUG**UGCCUUA** 3′	2844–2851
	hsa-miR-506-3p	3′ AGAUGAGUCUUCCC**ACGGAAU** 5′	
18	PABPC4L 3′ UTR	5′ CUUUUGUGCCCAAG**UGCCUUA** 3′	3244–3251
	hsa-miR-506-3p	3′ AGAUGAGUCUUCCC**ACGGAAU** 5′	
19	EZH2 3′ UTR	5′ UCCUCUGAAACAGC**UGCCUUA** 3′	36–42
	hsa-miR-506-3p	3′ AGAUGAGUCUUCCC**ACGGAAU** 5′	

Shown are (1) rank of the gene from HCS of neurite outgrowth, (2) gene name, (3) sequence and 3′UTR:miR-506-3p base pairing of each gene, and (4) the seed target sequence position in the 3′UTR for each gene.

**Table 2:** Correlation of mRNA levels with patient survival.

(1) Rank	(2) Gene	(3) Dataset	(4) OS Probability				(5) EFS Probability				(6) Oncogenic Potential
			Low Expression Group	High Expression Group	Raw *p* Value	Bonf *p* Value	Low Expresion Group	High Expression Group	Raw *p* Value	Bonf *p* Value	
1	*CDK4*	aKocak	0.85	0.41	1.29 × 10^−28^	5.95 × 10^−26^	0.75	0.33	4.90 × 10^−19^	2.26 × 10^−16^	**Yes**
		sSEQC	0.85	0.38	8.99 × 10^−36^	4.34 × 10^−33^	0.72	0.22	8.82 × 10^−28^	4.26 × 10^−25^	
2	*RAVER1*	aKocak	0.71	0.80	1.50 × 10^−2^	1	0.61	0.69	3.10 × 10^−2^	1	No
		sSEQC	0.85	0.27	1.85 × 10^−29^	8.91 × 10^−27^	0.70	0.25	1.66 × 10^−17^	8.01 × 10^−15^	
3	*SLC25A39*	aKocak	0.86	0.38	2.83 × 10^−24^	1.31 × 10^−21^	0.74	0.30	3.79 × 10^−16^	1.75 × 10^−13^	**Yes**
		sSEQC	0.89	0.40	3.50 × 10^−25^	1.69 × 10^−22^	0.75	0.32	2.28 × 10^−18^	1.10 × 10^−15^	
4	*ECI2*	aKocak	0.19	0.76	9.75 × 10^−11^	4.49 × 10^−8^	0.22	0.66	1.6 × 10^−4^	7.4 × 10^−2^	No
		sSEQC	0.47	0.76	6.95 × 10^−5^	3.40 × 10^−2^	0.40	0.63	5.10 × 10^−2^	1	
5	*SDF2L1*	aKocak	0.80	0.58	1.69 × 10^−5^	7.81 × 10^−3^	0.71	0.44	3.45 × 10^−7^	1.59 × 10^−4^	**Yes**
		sSEQC	0.79	0.45	9.27 × 10^−8^	4.48 × 10^−5^	0.66	0.30	2.72 × 10^−9^	1.32 × 10^−6^	
6	*RBL1*	aKocak	0.76	0.21	3.00 × 10^−11^	1.38 × 10^−8^	0.65	0.22	2.62 × 10^−4^	1.21 × 10^−1^	**Yes**
		sSEQC	0.78	0.21	4.52 × 10^−9^	2.18 × 10^−6^	0.64	0.27	8.19 × 10^−5^	4.00 × 10^−2^	
7	*SEPT9*	aKocak	0.26	0.76	8.96 × 10^−4^	4.13 × 10^−1^	0.46	0.65	3.40 × 10^−2^	1	No
		sSEQC	0.78	0.55	2.23 × 10^−6^	1.07 × 10^−3^	0.65	0.39	1.13 × 10^−4^	5.55 × 10^−2^	
8	*CNN3*	aKocak	0.80	0.59	1.68 × 10^−4^	7.7 × 10^−2^	0.69	0.50	7.45 × 10^−4^	3.43 × 10^−1^	**Yes**
		sSEQC	0.78	0.54	1.42 × 10^−5^	6.48 × 10^−3^	0.64	0.43	1.06 × 10^−3^	5.12 × 10^−1^	
9	*TMCO3*	aKocak	0.45	0.87	6.38 × 10^−16^	2.94 × 10^−13^	0.35	0.77	8.97 × 10^−15^	4.14 × 10^−12^	No
		sSEQC	0.48	0.87	5.30 × 10^−15^	2.56 × 10^−12^	0.42	0.71	1.32 × 10^−8^	6.37 × 10^−6^	
10	*MED20*	aKocak	0.76	0.63	1.20 × 10^−2^	1	0.66	0.47	2.37 × 10^−3^	1	**Yes**
		sSEQC	0.79	0.34	1.56 × 10^−9^	7.55 × 10^−7^	0.66	0.20	9.70 × 10^−13^	4.68 × 10^−10^	
11	*SURF4*	aKocak	0.81	0.55	1.36 × 10^−5^	6.27 × 10^−3^	0.71	0.44	3.29 × 10^−5^	1.5 × 10^−2^	**Yes**
		sSEQC	0.81	0.36	4.21 × 10^−20^	2.03 × 10^−17^	0.67	0.27	2.84 × 10^−14^	1.37 × 10^−11^	
12	*TCF3*	aKocak	0.79	0	9.85 × 10^−32^	4.54 × 10^−29^	0.68	0	1.75 × 10^−15^	8.09 × 10^−13^	**Yes**
		sSEQC	0.82	0.22	2.25 × 10^−34^	1.09 × 10^−31^	0.68	0.16	1.76 × 10^−19^	8.49 × 10^−17^	
13	*NFIB*	aKocak	0.83	0.52	2.02 × 10^−10^	9.29 × 10^−8^	0.69	0.49	1.18 × 10^−4^	5.4 × 10^−2^	**Yes**
		sSEQC	0.82	0.45	9.16 × 10^−14^	4.42 × 10^−11^	0.67	0.37	1.69 × 10^−8^	8.16 × 10^−6^	
14	*TUBB6*	aKocak	0.55	0.87	2.91 × 10^−15^	1.34 × 10^−12^	0.48	0.75	1.30 × 10^−8^	6.00 × 10^−6^	No
		sSEQC	0.80	0.53	1.41 × 10^−6^	6.80 × 10^−4^	0.67	0.34	6.41 × 10^−8^	3.10 × 10^−5^	
15	*SPDL1*	aKocak	0.77	0.40	1.25 × 10^−7^	5.75 × 10^−5^	0.67	0.28	6.92 × 10^−7^	3.19 × 10^−4^	**Yes**
		sSEQC	0.87	0.47	5.38 × 10^−17^	2.60 × 10^−14^	0.71	0.40	5.31 × 10^−13^	2.57 × 10^−10^	
16	*GATAD2A*	aKocak	0.81	0.39	2.75 × 10^−20^	1.27 × 10^−17^	0.70	0.34	4.08 × 10^−10^	1.88 × 10^−7^	**Yes**
		sSEQC	0.81	0.29	4.50 × 10^−27^	2.17 × 10^−24^	0.67	0.16	4.74 × 10^−18^	2.29 × 10^−15^	
17	*QKI*	aKocak	0.54	0.79	2.02 × 10^−4^	9.3 × 10^−2^	0.45	0.68	2.14 × 10^−3^	9.87 × 10^−1^	No
		sSEQC	0.81	0.64	2.09 × 10^−3^	1	0.65	0.53	1.70 × 10^−2^	1	
18	*PABP4CL*	aKocak	N/A	N/A	N/A	N/A	N/A	N/A	N/A	N/A	**Yes**
		sSEQC	0.77	0.25	6.58 × 10^−7^	3.18 × 10^−4^	0.63	0.27	5.30 × 10^−4^	2.56 × 10^−1^	
19	*EZH2*	aKocak	0.78	0.43	2.28 × 10^−4^	1.05 × 10^−1^	0.68	0.33	6.76 × 10^−7^	3.12 × 10^−4^	**Yes**
		sSEQC	0.80	0.46	1.48 × 10^−5^	7.15 × 10^−3^	0.65	0.43	1.33 × 10^−5^	6.43 × 10^−3^	

Shown are (1) the ranking of the genes from the HCS, (2) gene name, (3) patient dataset, (4) OS survival probability in the low- and high-expression groups and *p* value of the correlation, (5) EFS survival probability in the low- and high-expression groups and *p* value of the correlation, and (6) oncogenic potential as determined by both datasets. Yes means that a higher mRNA level is significantly correlated with poorer patient survival, which suggests the potential oncogenic function of the gene in neuroblastoma, whereas No means that a higher mRNA level is not significantly correlated with poorer patient survival.

## Data Availability

The original contributions presented in the study are included in the article, further inquiries can be directed to the corresponding author.

## Abbreviations

3′UTR, 3′ untranslated region; CDK4, cyclin-dependent kinase 4; CNN3, calponin 3; DMEM, Dulbecco’s Modified Eagle Medium; DMSO, dimethyl sulfoxide; ECI2, Enoyl-CoA Delta Isomerase 2; EFS, event-free survival; EZH2; enhancer of zeste 2 polycomb repressive complex 2 subunit; GATAD2A, GATA zinc finger domain-containing 2A; HCS, high-content screening; IPA, Ingenuity Pathway Analysis; MED20, mediator complex subunit 20; miRNAs, microRNAs; MTT, 2-(4,5-Dimethylthiazol-2-yl)-2,5-Diphenyltetrazolium Bromide; NFIB, nuclear factor I B; Oligo, oligonucleotides; OS, overall survival; PABPC4L, poly(A)-binding protein cytoplasmic 4 like; P/S, penicillin–streptomycin; QKI, KH domain-containing RNA binding; RAVER1, ribonucleoprotein; PTB binding 1; RBL1, retinoblastoma-like protein 1; SDF2L1, stromal cell-derived factor 2 like 1; SEPT9, septin 9; siRNA, small interfering RNA; SLC25A39, solute carrier family 25 member 39; SPDL1, spindle apparatus coiled-coil protein 1; SURF4, surfeit 4; TCF3, transcription factor 3; TMCO3, transmembrane and coiled-coil domains 3; TUBB6, tubulin β 6.
